# Cenobamate in super-refractory status epilepticus: current evidence from a patient-level synthesis

**DOI:** 10.3389/fneur.2026.1961349

**Published:** 2026-10-08

**Authors:** Giovanni Falcicchio, Teresa Francavilla

**Affiliations:** Department of Translational Biomedicine and Neuroscience (DiBraiN), University of Bari ‘Aldo Moro', Bari, Italy

**Keywords:** antiseizure medication (ASM), cenobamate, drug resistant epilepsy, status epilepticus, super refractory status epilepticus

## Abstract

**Background:**

Super-refractory status epilepticus (SRSE) represents a challenging neurological emergency, with limited therapeutic options after conventional intravenous antiseizure medications (ASMs) and continuous anesthetic therapy have failed. Cenobamate has shown remarkable efficacy in drug-resistant focal epilepsy, but its role in SRSE is currently supported only by isolated case reports.

**Methods:**

We performed a patient-level synthesis of published cases of cenobamate-treated SRSE, focusing on treatment latency, titration strategy, electroclinical response, concomitant therapies, safety, and functional outcomes.

**Results:**

Three publications reporting six patients were identified. Cenobamate was introduced between 13 and 103 days after SRSE onset, following failure of multiple intravenous and oral ASMs, anesthetic agents, and, in most cases, immunomodulatory therapies. Although the timing of treatment initiation varied substantially, electroencephalographic improvement generally occurred within two weeks after cenobamate initiation and was usually accompanied by clinical recovery, successful withdrawal of anesthetic therapy, and liberation from the intensive care unit. Most patients initiated cenobamate at the recommended dose of 12.5 mg/day but underwent individualized dose-escalation schedules compared with the approved outpatient regimen. No cases of drug reaction with eosinophilia and systemic symptoms, severe cardiac adverse events, or treatment discontinuation attributable to cenobamate were reported.

**Conclusions:**

Although the available evidence remains limited to a small number of observational reports, this patient-level synthesis identified consistent clinical patterns that support further investigation of cenobamate as an adjunctive therapy for SRSE. At present, no conclusions regarding treatment efficacy can be drawn. Prospective multicenter studies are needed to clarify cenobamate optimal timing, titration strategy, safety, and potential role within SRSE treatment algorithms.

## 1 Background

Status epilepticus (SE) is a neurological emergency requiring prompt recognition and treatment to reduce morbidity and mortality (1). Early administration of antiseizure medications (ASMs) is essential to terminate ongoing seizure activity, prevent severe metabolic disturbances, and minimize long-term neurological sequelae (1). Selecting the most appropriate ASM in the acute setting remains challenging, as each drug has specific pharmacokinetic properties, efficacy profiles, potential adverse effects, and drug–drug interactions that must be carefully considered, particularly in critically ill patients or those with significant comorbidities (1).

In most patients, SE resolves following the timely administration of a benzodiazepine followed by one or more second-line ASMs (1). When seizures persist despite adequate first- and second-line treatment, the condition is defined as refractory status epilepticus (RSE), requiring the initiation of continuous intravenous anesthetic therapy. If seizures continue or recur 24 h or more after the onset of anesthetic treatment—including recurrence during anesthetic tapering or withdrawal—the condition is classified as super-refractory status epilepticus (SRSE). SRSE represents one of the most severe forms of SE and is associated with substantial morbidity, prolonged intensive care unit (ICU) stay, and poor functional outcomes, with an in-hospital mortality approaching 25% and fewer than 10% of patients achieving functional independence at hospital discharge (2).

Therapeutic options for SRSE remain limited, and evidence supporting the use of adjunctive oral ASMs in this setting is largely restricted to small case series and observational reports (2, 3). Consequently, no evidence-based recommendations currently exist regarding the optimal timing, dosing strategy, or patient selection for these therapies.

Cenobamate, a novel ASM with a unique dual mechanism of action combining preferential inhibition of persistent sodium currents and positive allosteric modulation of GABA_A_ receptors, has emerged as one of the most effective treatments for drug-resistant focal epilepsy, with seizure freedom reported in up to one-third of treated patients (4). These pharmacological properties make cenobamate an attractive candidate for the treatment of SRSE, a condition characterized by progressive pharmacoresistance and altered inhibitory and excitatory neurotransmission. Nevertheless, experience with cenobamate in the ICU setting remains limited. One potential barrier to its wider use is the recommended “start low, go slow” titration schedule, which was developed to minimize the risk of drug reaction with eosinophilia and systemic symptoms (DRESS) but may be difficult to reconcile with the urgent therapeutic needs of patients with SRSE (3).

The aim of the present Mini Review is to summarize the available patient-level evidence on the use of cenobamate in SRSE. Emphasis was placed on treatment timing, titration strategies, electroclinical response, concomitant therapies, safety, and functional outcomes to provide a clinically oriented synthesis of the currently available evidence.

## 2 Methods

Given the limited published evidence regarding the use of cenobamate in SRSE, a focused literature review followed by a patient-level evidence synthesis was performed.

The literature search was conducted in PubMed, Scopus, and Web of Science, with the final search performed on July 31, 2026. The following database-specific search strategies were used: PubMed: (“cenobamate”[Title/Abstract] OR “YKP3089”[Title/Abstract]) AND (“status epilepticus”[Title/Abstract] OR “refractory status epilepticus”[Title/Abstract] OR “super-refractory status epilepticus”[Title/Abstract] OR “NORSE”[Title/Abstract] OR “FIRES”[Title/Abstract]); Scopus: TITLE-ABS-KEY(cenobamate OR YKP3089) AND TITLE-ABS-KEY(“status epilepticus” OR “refractory status epilepticus” OR “super-refractory status epilepticus” OR NORSE OR FIRES); and Web of Science: TS=(cenobamate OR YKP3089) AND TS=(“status epilepticus” OR “refractory status epilepticus” OR “super-refractory status epilepticus” OR NORSE OR FIRES). No restrictions on publication date or language were applied. Studies conducted exclusively in non-human subjects were excluded during screening. Reference lists of eligible articles were manually screened to identify additional relevant reports.

Studies were considered eligible if they fulfilled all the following criteria:

(i) human subjects;
(ii) diagnosis of SRSE;
(iii) administration of cenobamate during the active phase of SE or as part of the therapeutic strategy aimed at achieving electroclinical resolution; and
(iv) availability of sufficient patient-level clinical information for data extraction. Publications were excluded if they described drug-resistant epilepsy without SE, represented narrative reviews without original clinical observations, consisted solely of conference abstracts or reports lacking adequate clinical detail, or duplicated previously published patient data.

Because the available evidence consisted exclusively of isolated case reports, small case series, and letters to the editor, patient-level data extraction was considered the most appropriate strategy to maximize comparability across studies. Consequently, all publications reporting original patient-level clinical data were included irrespective of publication format, provided that sufficient clinical information was available.

For each patient, the following variables were extracted whenever available: age, sex, previous history of epilepsy, etiology of SE, SE phenotype (i.e., convulsive, non-convulsive), magnetic resonance imaging (MRI), electroencephalographic (EEG) pattern (e.g., periodic discharges, rhythmic delta activity or spike-and-wave/sharp-and-wave patterns), duration of SRSE before cenobamate initiation, previous ASMs, intravenous anesthetic and immunomodulatory therapies, other adjunctive treatments (e.g., ketogenic diet and vagus nerve stimulation), cenobamate starting dose and titration strategy, time to EEG improvement and time to clinical response, adverse events, SE termination and functional outcome (ICU liberation, discharge outcome and follow-up when available).

Given the small number of available cases and the marked heterogeneity of patient characteristics, treatment strategies, and outcome measures, quantitative meta-analysis was neither feasible nor appropriate. Therefore, data were synthesized descriptively through a comparative patient-level analysis. To facilitate interpretation, findings were organized into five predefined clinically relevant domains: (i) therapeutic latency, (ii) cenobamate titration strategy, (iii) electroclinical response, (iv) concomitant treatment map, and (v) safety under polytherapy.

## 3 Results

### 3.1 Literature search

The literature search identified three publications reporting original patient-level clinical data on the use of cenobamate in SRSE (3, 5, 6). A total of six patients were included in the comparative patient-level analysis.

### 3.2 Baseline patient characteristics

Baseline demographic and clinical characteristics of the six patients included in the patient-level analysis are summarized in Table 1.

**Table 1:** Characteristics of the included reports and baseline demographic and clinical features before cenobamate initiation.

Study characteristics	Patient	Age/sex	Previous epilepsy	SE phenotype	Etiology	EEG pattern	MRI	Previous ASMs (n)	Intravenous anesthetics	Immunotherapy	Other treatments
***Carlson et al**. **(****6****); USA; single-center case series; n** **=** **2***	Case 1	45/M	No	Convulsive	FIRES	Frontal sharp waves evolving to SE	Nonspecific leptomeningeal FLAIR hyperintensity	8	Propofol, midazolam, ketamine, pentobarbital	MP, IVIG, rituximab	Ketogenic diet, antibiotics
	Case 2	52/F	No	Focal evolving to NCSE	HSV-1 encephalitis	Right temporal LPDs	T2/FLAIR hyperintensity in the right insular and left medial temporal cortex	8	Propofol, midazolam	IVIG	Ketogenic diet, acyclovir, decompressive hemicraniectomy, temporal lobectomy
***Falcicchio et al**. **(****5****); Italy; single-patient report; n=1***	-	26/F	No	NCSE	Seronegative autoimmune encephalitis	Diffuse slowing	T2/FLAIR hyperintense lesion in the right occipital lobe	5	Propofol, midazolam, thiopental	MP, IVIG, PLEX, tocilizumab	Antibiotics, acyclovir
***Romozzi et al**. **(****3****); Italy; retrospective case series; n** **=** **3***	Case 1	28/M	No	Convulsive	Unknown	Right hemispheric rhythmic discharges (2–3 Hz)	Bilateral mesial temporal, insula, claustrum, and pulvinar T2/FLAIR hyperintensities	6	Propofol, midazolam, ketamine	MP, IVIG, PLEX, tocilizumab	Ketogenic diet, antibiotics
	Case 2	75/F	No	NCSE	Unknown	Generalized spike-and-wave (2.5–3 Hz)	Bilateral hemispheric FLAIR hyperintensities and right frontal leptomeningeal changes	3	Propofol	MP, IVIG	Antibiotics, acyclovir
	Case 3	27/F	No	Convulsive	FIRES	Generalized ictal discharges	Mesial temporal hyperintensities	7	Propofol, ketamine	MP, IVIG, PLEX	Ketogenic diet, antibiotics

Report-level characteristics and baseline demographic, electroclinical, and therapeutic features of the six patients included in the patient-level evidence synthesis are reported as extracted from the original publications (3, 5, 6). Previous ASMs refers to the number of ASMs administered before cenobamate introduction. Immunotherapy includes corticosteroids, intravenous immunoglobulins, plasma exchange, rituximab, tocilizumab, or other immunomodulatory therapies according to the presumed underlying etiology. FIRES, febrile infection-related epilepsy syndrome; IVIG, intravenous immunoglobulins; LPDs, lateralized periodic discharges; MP, methylprednisolone; NCSE, non-convulsive status epilepticus; PLEX, plasma exchange; SE, status epilepticus.

Patients ranged in age from 26 to 75 years (median 36.5 years), with a female predominance (4/6). None had a previously documented history of epilepsy before the onset of the condition leading to SRSE. Clinical presentation included both convulsive (3/6) and non-convulsive (2/6) status epilepticus, while one patient initially presented with focal seizures evolving to NCSE. Etiologies were heterogeneous and comprised FIRES (*n* = 2), viral encephalitis (*n* = 1), seronegative autoimmune encephalitis (*n* = 1), cryptogenic/unknown etiologies (*n* = 2).

Neuroimaging findings were abnormal in all patients and reflected the underlying disease process.

EEG findings were similarly heterogeneous, ranging from focal rhythmic or ictal discharges to generalized spike-and-wave activity, lateralized periodic discharges, and diffuse background slowing.

Before cenobamate initiation, all patients had received multiple ASMs (median 6.5 ASMs, range 3–8) together with prolonged intravenous anesthetic therapy. Propofol was administered in all patients, midazolam in four, ketamine in three, and barbiturates in three. Immunomodulatory treatment preceded cenobamate administration in all patients and most commonly included intravenous methylprednisolone and intravenous immunoglobulins; additional treatments included plasma exchange, rituximab, and tocilizumab according to the presumed underlying etiology and clinical course. Adjunctive treatments included ketogenic diet (*n* = 4), antibiotics (*n* = 5), and acyclovir (*n* = 3). One patient with HSV-1 encephalitis underwent decompressive hemicraniectomy followed by temporal lobectomy.

### 3.3 Patient-level therapeutic timeline

To facilitate direct comparison of treatment trajectories across the available evidence, a patient-level therapeutic timeline was constructed for all cases with sufficient chronological information (Figure 1). Patients lacking adequate temporal information to reconstruct the complete therapeutic sequence were not included in the timeline but remained part of the overall patient-level evidence synthesis and were considered in the analyses of treatment strategies and safety (5).

**Figure 1:**
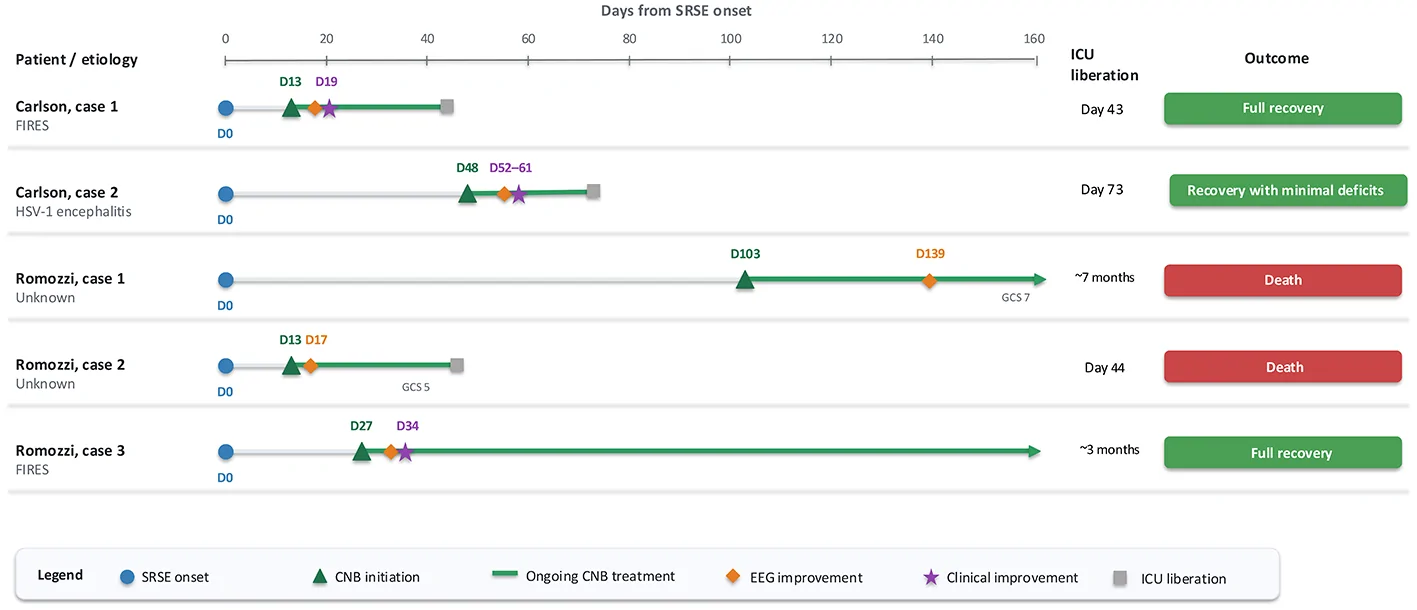
Patient-level clinical evidence map of cenobamate-treated super-refractory status epilepticus. The figure summarizes the therapeutic trajectory of each evaluable patient from SRSE onset (Day 0) to functional outcome. For each case, the timing of cenobamate initiation, electroencephalographic improvement, clinical improvement, ICU liberation, and final outcome are displayed on a common temporal scale, allowing direct comparison of treatment latency and response patterns across patients. The visualization emphasizes the considerable variability in the timing of cenobamate introduction despite a relatively homogeneous interval between treatment initiation and electroclinical improvement in most cases. Patients without sufficient temporal information were excluded from the graphical representation but were included in the overall evidence synthesis. CNB, cenobamate; EEG, electroencephalography; GCS, Glasgow Coma Scale; SRSE, super-refractory status epilepticus.

The interval between SRSE onset and cenobamate initiation ranged from 13 to 103 days, indicating substantial variability in treatment latency across the published cases. Cenobamate was generally administered using individualized titration schedules that were faster than the standard outpatient regimen recommended for drug-resistant focal epilepsy, reflecting the urgency of seizure control in the ICU setting. Notably, all but one patient started treatment at the recommended initial dose of 12.5 mg/day; the only exception received 100 mg/day as the starting dose (5). Subsequent dose escalation was consistently tailored according to the patient's clinical condition, with final maintenance doses ranging from 100 to 400 mg/day, although 200 mg/day was the most commonly achieved dose (Table 2).

**Table 2:** Comparison of cenobamate titration strategies in patients with super-refractory status epilepticus.

Study (patient)	Starting dose	Dose-escalation schedule	Maximum dose	Titration approach
*Carlson*, *case 1*	12.5 mg/day	12.5 mg × 7 d → 25 mg × 1 d → 50 mg, with subsequent escalation to 200 mg/day	200 mg/day	Accelerated
*Carlson*, *case 2*	12.5 mg/day	12.5 mg × 9 d → 25 mg × 7 d → 50 mg, with subsequent escalation to 200 mg/day	200 mg/day	Accelerated
*Romozzi*, *case 1*	12.5 mg/day	12.5 mg × 14 d → 25 mg × 14 d → 50 mg × 14 d → 100 mg × 14 d → 150 mg × 14 d → 200 mg, then increased to 400 mg/day	400 mg/day	Accelerated, prolonged escalation
*Romozzi*, *case 2*	12.5 mg/day	12.5 mg × 14 d → 25 mg × 14 d → 50 mg × 14 d → 100 mg/day	100 mg/day	Accelerated
*Romozzi*, *case 3*	12.5 mg/day	12.5 mg × 7 d → 25 mg × 7 d → 50 mg × 14 d → 100 mg × 14 d → 150 mg × 28 d → 200 mg/day	200 mg/day	Individualized
*Falcicchio*	100 mg (loading dose)	Loading dose followed by maintenance titration to 150 mg/day after seizure recurrence	150 mg/day	Loading strategy

The table summarizes the initial dose, dose-escalation schedule, maximum dose, and overall titration strategy reported in the available literature and in the present case. Most patients started cenobamate at 12.5 mg/day and underwent accelerated or individualized titration, whereas one patient received a 100 mg loading dose. The report by Falcicchio et al. was included exclusively for comparison of titration strategy.

Despite the individualized titration strategies, no severe adverse events were reported across the included cases. In particular, no cases of DRESS, severe cardiac adverse events, or treatment discontinuation due to cenobamate toxicity were described. Reported adverse events were limited to isolated and generally manageable pharmacological interactions or dose adjustments.

Despite the marked variability in the timing of cenobamate initiation, EEG improvement was observed within two weeks of treatment initiation in most patients. The only exception was one patient reported by Romozzi et al., who showed improvement approximately 36 days after cenobamate initiation (3). Whenever reported, clinical improvement followed a similar temporal pattern and was generally associated with successful withdrawal of intravenous anesthetic therapy and liberation from the ICU. ICU liberation was achieved in all reported cases, although its timing varied substantially, ranging from 43 days to approximately 7 months after SRSE onset. Functional outcomes remained heterogeneous, ranging from complete neurological recovery and return to normal daily activities to persistent neurological disability or death related to systemic complications.

## 4 Discussion

In SRSE enterally administered ASMs may represent a valuable adjunctive strategy when conventional intravenous therapies have failed or when a sustained non-anesthetic regimen is required to facilitate the withdrawal of continuous anesthetic infusions (7). Their principal advantage lies in enabling the introduction of agents with complementary mechanisms of action that are unavailable in intravenous formulations, thereby broadening therapeutic options and providing a pharmacological bridge during withdrawal of anesthetic therapy (7). Although the evidence supporting this approach remains predominantly observational, one potential rationale for introducing additional non-anesthetic ASMs is to facilitate withdrawal of continuous anesthetic agents and thereby limit exposure to their hemodynamic and respiratory adverse effects. Whether this strategy improves clinical outcomes, however, remains uncertain.

Within this therapeutic framework, cenobamate represents a particularly attractive candidate because of its dual mechanism of action. In addition to preferential inhibition of persistent sodium currents, cenobamate acts as a positive allosteric modulator of GABA_A_ receptors through a binding site distinct from that of benzodiazepines. Theoretically, these complementary mechanisms target several of the neurobiological processes implicated in pharmacoresistant SE, including impaired GABAergic inhibition, persistent sodium channel activation, and sustained glutamatergic hyperexcitability (3).

To our knowledge, this study provides the first patient-level synthesis specifically focusing on the use of cenobamate in patients with SRSE. Although the available evidence remains limited to a small number of patients, the comparative analysis identified some recurring patterns regarding the timing of cenobamate introduction, titration strategies, electroclinical response, and safety. These observations may help contextualize the potential role of cenobamate within the therapeutic algorithm of SRSE while highlighting the current evidence gaps.

### 4.1 Therapeutic latency

One of the most striking findings emerging from the available cases is the remarkable heterogeneity in the timing of cenobamate initiation. Across the published reports, cenobamate was introduced between day 13 and day 103 after SRSE onset, reflecting the absence of standardized recommendations regarding the optimal timing for its use.

This heterogeneity in the timing of cenobamate initiation likely reflects its current off-label role in the management of SRSE. While cenobamate has become an established treatment for drug-resistant focal epilepsy, supported by robust clinical trials and extensive real-world evidence (8), its use in SRSE is still limited to anecdotal clinical experience. In the absence of dedicated recommendations or standardized treatment algorithms, the decision to introduce cenobamate is largely physician-dependent, resulting in considerable variability in the timing of administration across the published cases.

Another possible explanation for the delayed introduction of cenobamate may be the complexity of pharmacological management in patients with SRSE. Because these patients are typically treated with multiple concomitant medications—including ASMs, anesthetic agents, antimicrobials, and other intensive care therapies—clinicians may understandably adopt a cautious approach when considering the addition of cenobamate, given its potential for pharmacokinetic interactions. Consequently, in the context of SRSE cenobamate is generally introduced only after multiple intravenous ASMs, continuous anesthetic infusions and, in many cases, immunomodulatory therapies have been attempted. Whether earlier administration could improve seizure control, facilitate anesthetic withdrawal or shorten ICU stay remains completely unknown.

The temporal heterogeneity observed across the available cases therefore emphasizes the need for prospective studies specifically evaluating the optimal therapeutic window for cenobamate initiation during SRSE.

### 4.2 Cenobamate titration strategy

Another important observation emerging from the patient-level comparison concerns the cenobamate titration strategies adopted in critically ill patients with SRSE.

As summarized in Table 2, all but one patient initiated cenobamate at the recommended starting dose of 12.5 mg/day, whereas only a single case received a 100 mg loading dose (5). Rather than increasing the initial dose, clinicians generally adopted individualized dose-escalation schedules, reflecting the need to balance the urgency of seizure control against the potential safety concerns associated with cenobamate use in critically ill patients.

Compared with the standard outpatient titration regimen recommended for drug-resistant focal epilepsy, dose escalation was substantially fast in all reported SRSE cases, although the degree of acceleration varied according to the patient's clinical condition and the treating physician's judgment.

In this setting, accelerated titration may be clinically attractive because the conventional outpatient schedule is poorly suited to the time-sensitive nature of SRSE, in which prolonged seizure activity and continued exposure to anesthetic agents may independently contribute to morbidity.

Despite these individualized and frequently accelerated titration schedules, no cases of DRESS or other severe cutaneous adverse reactions have been reported to date. Reported adverse events were generally mild or manageable and consisted mainly of somnolence, gastrointestinal symptoms, or pharmacokinetic interactions requiring adjustment of concomitant ASMs, including phenytoin toxicity that necessitated immediate phenytoin withdrawal (5).

Nevertheless, interpretation of these findings is limited by the extremely small number of treated patients, the retrospective nature of the available evidence, and the concomitant administration of multiple antiseizure and anesthetic agents, which makes it difficult to attribute both efficacy and toxicity to cenobamate alone.

Overall, the available observations suggest that accelerated cenobamate titration may be feasible in carefully selected patients with SRSE when performed under intensive clinical, EEG, and laboratory monitoring. However, these preliminary findings are insufficient to redefine current safety recommendations. Prospective studies are needed to clarify the pharmacokinetics of enterally administered cenobamate in critically ill patients and to determine the optimal balance between rapid dose escalation, seizure control, drug interactions, and treatment safety.

### 4.3 Electroclinical response

An informative pattern emerging from the patient-level comparison concerns the temporal relationship between EEG and clinical recovery. In all cases for which both outcomes were adequately reported, EEG improvement occurred concurrently with, or within the same reported time window as, the first signs of clinical improvement. This close temporal association suggests that electrographic seizure control may represent the earliest objectively measurable component of treatment response, although it should not be regarded as equivalent to meaningful neurological or functional recovery.

This distinction is particularly relevant in SRSE, where prolonged exposure to anesthetic agents, critical illness, mechanical ventilation, and ICU-acquired weakness frequently limit the reliability of the neurological examination. In this context, continuous EEG monitoring remains essential for documenting seizure termination, assessing treatment response, and supporting the progressive withdrawal of intravenous anesthetic therapy. Across the available cases, electrographic improvement was consistently followed by successful anesthetic weaning and ICU liberation, although subsequent neurological outcomes remained markedly heterogeneous.

Importantly, electrographic response did not invariably predict favorable functional outcome. One patient showed EEG improvement without a documented clinical response, while another achieved seizure termination but subsequently died from systemic complications. Therefore, EEG improvement should be interpreted as an early marker of seizure control rather than as a surrogate for long-term neurological recovery. Although causality cannot be established from uncontrolled observations, the reproducible temporal association between EEG improvement, anesthetic withdrawal, and initial clinical recovery supports further investigation of continuous EEG-derived endpoints as early response markers in cenobamate-treated SRSE.

### 4.4 Concomitant-treatment map

Interpretation of the therapeutic contribution of cenobamate should be undertaken with appropriate caution, as all reported patients received complex multimodal treatment throughout the course of SRSE. Before cenobamate initiation, patients had already failed multiple intravenous and oral ASMs, prolonged anesthetic infusions, and, in most cases, additional interventions including immunotherapy, ketogenic diet, antimicrobial therapy, or other etiology-specific treatments. Consequently, the eventual resolution of SE cannot be unequivocally attributed to cenobamate alone.

Rather than representing an isolated rescue therapy, cenobamate should currently be viewed as one component of a comprehensive multimodal treatment strategy. Nevertheless, an interesting observation emerging from the patient-level comparison is that, despite the remarkable heterogeneity of the preceding therapeutic approaches, the clinical trajectory following cenobamate initiation appeared relatively consistent across the available cases. In most evaluable patients, electrographic improvement was followed by successful withdrawal of continuous anesthetic therapy, liberation from the ICU, and subsequent clinical recovery, although long-term functional outcomes remained highly variable.

Although these observations do not establish a causal relationship, they raise the hypothesis that cenobamate may contribute to seizure control once conventional therapeutic strategies have proven ineffective, possibly facilitating the transition from prolonged anesthetic-dependent management to maintenance antiseizure therapy. This hypothesis is biologically plausible considering cenobamate's dual mechanism of action, combining preferential inhibition of persistent sodium currents with positive allosteric modulation of GABA_A_ receptors, although its specific contribution within the multimodal treatment of SRSE remains impossible to quantify from the currently available evidence.

Future investigations should therefore focus not only on determining whether cenobamate is effective in SRSE, but also on identifying the optimal timing for its introduction, the most appropriate titration strategy, and its integration into standardized multimodal treatment algorithms. Prospective multicenter registries and collaborative studies will be essential to clarify the independent contribution of cenobamate while accounting for the numerous concomitant therapies that characterize the management of this challenging condition.

### 4.5 Safety under polytherapy

Safety represents one of the major concerns when introducing a novel ASM in critically ill patients already exposed to extensive polytherapy (9). Beyond the recognized risk of DRESS, particularly associated with rapid initiation, cenobamate may interact pharmacokinetically with several concomitant ASMs through inhibition of CYP2C19 and induction of CYP3A4 and CYP2B6, potentially increasing serum concentrations of drugs such as phenytoin and phenobarbital while reducing exposure to others, including carbamazepine and lamotrigine (10). Consequently, careful therapeutic monitoring and individualized dose adjustment are recommended, particularly in the ICU setting where patients frequently receive multiple ASMs together with anesthetic agents, antimicrobials, and other concomitant medications.

Large clinical trials and subsequent real-world studies conducted in patients with drug-resistant focal epilepsy have consistently demonstrated a favorable tolerability profile for cenobamate, with dizziness, somnolence, fatigue, and diplopia representing the most commonly reported adverse events (11, 12). Importantly, the implementation of the current ‘start low, go slow' titration schedule has dramatically reduced the incidence of DRESS, which has become exceedingly rare in routine clinical practice (10). These observations have contributed to the widespread adoption of cenobamate in the management of focal epilepsy, although they derive from outpatient populations that differ substantially from patients with SRSE.

Despite the substantially more complex therapeutic setting and the use of accelerated or individualized titration schedules, the currently available SRSE literature has not identified major safety signals attributable to cenobamate. Across the published cases, no episodes of DRESS, severe cardiac adverse events, or treatment discontinuation due to cenobamate toxicity have been reported. Adverse events were limited to transient somnolence in two patients, gastrointestinal symptoms in one patient and one episode of phenytoin toxicity, which was consistent with the known CYP2C19-mediated pharmacokinetic interaction between the two drugs rather than an intrinsic toxic effect of cenobamate itself. Overall, these findings suggest that, under close ICU monitoring and appropriate management of concomitant therapies, cenobamate appears to be reasonably well tolerated even in highly complex pharmacological settings.

Nevertheless, these observations should be interpreted with caution. The available evidence is based on a very limited number of patients, heterogeneous reporting of adverse events, and relatively short follow-up. Rare complications may therefore remain undetected, and the true incidence of clinically relevant drug–drug interactions in critically ill patients is still unknown. As clinical experience with cenobamate in SRSE expands, prospective pharmacovigilance studies and systematic reporting of adverse events will be essential to better define its safety profile and optimize its integration into multimodal treatment strategies.

Taken together, these patient-level observations suggest that the current clinical strategy for cenobamate use in SRSE differs from its approved use in drug-resistant focal epilepsy. Rather than increasing the initial dose, clinicians consistently preserved the recommended starting dose while individualizing and accelerating dose escalation according to the patient's clinical course. Whether earlier initiation and standardized accelerated titration could further improve outcomes without compromising safety remains an important unanswered question deserving prospective investigation.

## 5 Strengths and limitations

The main strength of this study lies in the utilization of a structured patient-level synthesis of all currently available reports, allowing direct comparison of therapeutic timing, electroclinical evolution, and clinical outcomes. This approach provides a more detailed overview than narrative descriptions alone and identifies common patterns that may not be apparent from individual case reports.

Several limitations should also be acknowledged. The available evidence is restricted to a very small number of heterogeneous cases, all derived from uncontrolled observational reports. Treatment strategies, timing of cenobamate initiation, concomitant therapies, EEG reporting, and outcome assessment differed considerably across studies, limiting direct comparisons. Furthermore, the absence of control groups precludes any causal inference regarding treatment efficacy.

Moreover, the available evidence is also particularly susceptible to publication and survivor bias. Consequently, the response rates and clinical outcomes observed in this patient-level synthesis may overestimate the potential benefit of cenobamate and should not be interpreted as representative of the broader SRSE population.

## 6 Conclusions

Current evidence regarding the use of cenobamate in SRSE remains limited but suggests that the drug may represent a promising adjunctive therapeutic option in selected patients with prolonged refractory seizures. The patient-level analysis identified consistent patterns of electroclinical improvement following cenobamate introduction, while accelerated titration appeared feasible under intensive monitoring without major safety concerns in the published cases. However, the available evidence remains insufficient to establish efficacy or define the optimal timing of administration. Prospective multicenter studies with standardized treatment protocols are warranted to clarify the role of cenobamate within the management of SRSE.
